# Pathogenesis, diagnosis, dietary management, and prevention of gastrointestinal disorders in the paediatric population

**DOI:** 10.1186/s13052-022-01366-8

**Published:** 2022-09-11

**Authors:** Giulio Pulvirenti, Vincenzo Sortino, Sara Manti, Giuseppe Fabio Parisi, Maria Papale, Alessandro Giallongo, Salvatore Leonardi

**Affiliations:** 1 Pediatric Respiratory and Cystic Fibrosis Unit, Department of Clinical and Experimental Medicine, San Marco Hospital, University of Catania, Via Santa Sofia 78, 95123 Catania, Italy; 2 Pediatric Unit, Department of Human and Pediatric Pathology “Gaetano Barresi”, AOUP G. Martino, University of Messina, Via Consolare Valeria, 1, 98124 Messina, Italy

**Keywords:** Diet, Children, Food allergy, Cystic fibrosis, Gastroesophageal reflux, Inflammatory bowel disease, Short-bowel syndrome, Eosinophilic esophagitis

## Abstract

Nutrition has a central role in child growth with long-term effects, and nutrition management in gastrointestinal disorders has great importance for child health and disease outcomes. Breast milk is the first choice for infant nutrition. When it is not available, special milk formulas are adopted in specific conditions, as a medical treatment. Moving from the strong guidelines, recommendations and the new possibilities of special diet treatment, this review will analyse the current diet treatment in different gastrointestinal disorders, including food allergy, cystic fibrosis, inflammatory bowel diseases, short-bowel syndrome, gastroesophageal reflux, and eosinophilic esophagitis. The review also aimed at understanding the role of diet and its effects on these diseases. The growth monitoring can prevent malnutrition and improve disease outcomes, particularly in children, and an appropriate dietary management targeted to specific disorders is the best therapeutic choice alone or in combination with pharmacological therapy.

## Introduction

According to the World Health Organization (WHO), “Nutrition is a critical part of health and development” [1]. During the first infancy, correct nutrition can influence the lifelong health of the human being. Specifically, whether good nutrition is crucial for health status, poor nutrition, such as undernutrition or overnutrition, can increase vulnerability to preventable diseases and favour physical and mental disorders. On the other hand, the pathological mechanisms underlying several diseases may be modified by specific bioactive compounds of foods that can prevent disease development, influence disease progression, representing a potential treatment. Accordingly, great interest has grown in nutrition management in some pathological conditions, including gastrointestinal disorders.

Herein, we aim to provide an updated overview of observational and intervention studies investigating the effects of diet on gastrointestinal disorders, such as food allergy (FA), cystic fibrosis (CF), inflammatory bowel diseases (IBD), short-bowel syndrome (SBS), gastroesophageal reflux disease (GERD), and eosinophilic esophagitis (EoE).

## Methods

**Fig. 1:**
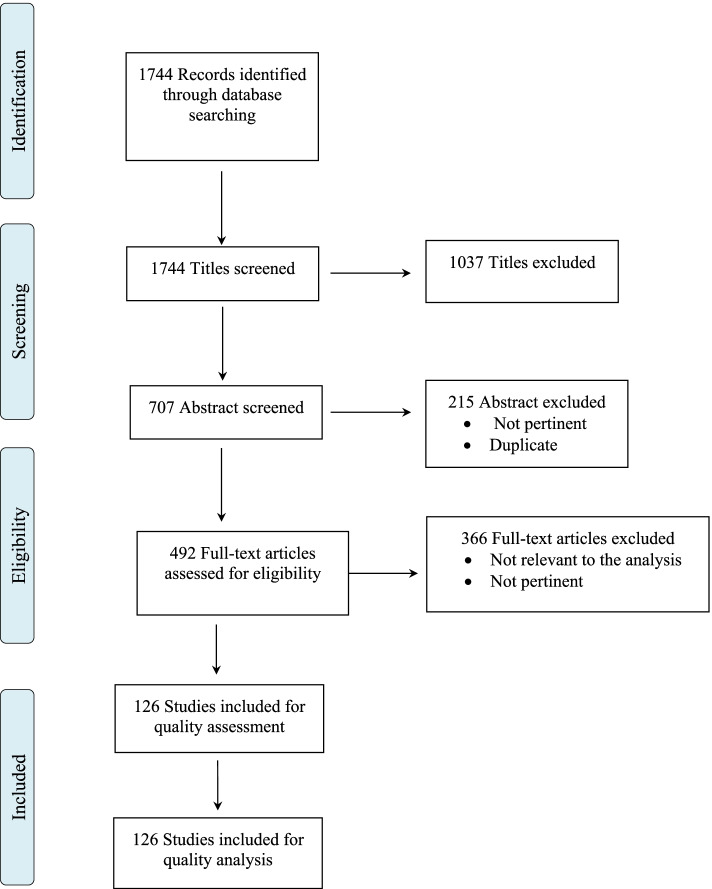
Flow chart of the literature research

This review was conducted using the international database PubMed. From this website, we have searched for articles in English using the following keywords: (1) special diet; (2) gastrointestinal disorders; (3) diet treatment and gastroesophageal reflux disease; (4) diet treatment and cystic fibrosis; (5) diet and IBD; (6) diet and short-bowel syndrome; and (7) diet and eosinophilic esophagitis. We have chosen mainly studies based on child cohorts, and we have extracted evidence from the studies of the last ten years (Fig. 1). We have used the article’s abstracts to decide if the articles were of interest to the topic. We also have reviewed the references of the selected articles and read those that might interest the current topic.

### Diet management in food allergy

FA is a common disorder among children; its incidence is estimated at around 8%. FA is an adverse immune response to food proteins, and results in gastrointestinal and extra-intestinal symptoms, affecting the skin, with erythematous rashes, pruritus, urticaria, and angioedema; upper or lower airways; cardiovascular and nervous systems. There is also the possibility of generalized reactions when there is a severe immune response to some allergens. The diagnosis of FA is based on the clinical history and laboratory findings, such as in vivo (skin prick test) and in vitro (allergen-specific serum immunoglobulin (Ig)E) tests, and elimination diet. The gold standard for diagnosis is the oral food challenge [2, 3].

FA is classified into non-IgE-mediated disease and IgE-mediated disease, but there are also mixed forms, with clinical manifestations which could be heterogeneous, involving different organs like skin, gut or airways. Food protein-induced enterocolitis syndrome, food protein-induced enteropaty, food protein-induced proctocolitis, and food protein-induced hemosiderosis must also be included in the differential diagnosis [4, 5].

Non-IgE-mediated FAs are caused by an immune response involving other components apart from IgE antibodies. The reactions do not appear immediately after the ingestion of the food and commonly involve the skin and gastrointestinal tract, causing also enterocolitis syndrome and rectocolitis [4–7].

Two adjacent IgE molecules, binding to the food protein, lead to the degranulation of the mast cell and basophil with a consequent release of preformed mediator within minutes of exposure. This type of reaction is immediate and generally occurs within 2 h after the ingestionIt can consist of acute urticaria, angioedema, vomiting, and wheezing. There is the possibility for late manifestations, with described reactions one week after milk ingestion. The presence of specific serum IgE can support the immune sensitization in IgE-mediated allergy, and the specific IgE levels seem to predict the severity of the manifestations [4, 5].

Among a large range of immunogenic foods, eight foods only provoke 90% of IgE-mediated food-allergic reactions: cow’s milk, egg, soy, wheat, shellfish, fish, peanuts, and tree nuts (Table 1) [8]. Cow's milk proteins allergy (CMA) is the most common cause of FA in the paediatric population, especially under the age of 3 years [9, 10], with a prevalence of 2–3% [11]. CMA is the consequence of ingesting cow’s milk proteins, which are particularly immunogenic in susceptible children. The clinical patient’s history provides the elements to suppose a diagnosis. The major allergenic proteins in cow’s milk are casein proteins: αs1-, αs2-, β- and κ-casein; and whey proteins: α-lactalbumin and β-lactoglobulin [11].

**Table 1:** The eight types of foods causing 90% of IgE-mediated food-allergy reactions

Food	Cow’s milk	Egg	Soy	Wheat	Fish	Shellfish	Peanuts	Tree nuts
**Allergenic proteins**	αs1- αs2- β- and κ-casein, α-lactalbumin β-lactoglobulin	ovomucoid (Gal d 1), ovalbumin (Gal d 2), ovotransferrin (Gal d 3) and lysozyme (Gal d 4)	Gly m1 to Gly m6. ß-conglycinin (Gly m5) is considered the main allergen in childhood	glutelins and prolamins	parvalbumin	Tropomyosin, Arginine kinase, Myosin light chain, Sarcoplasmic calcium binding protein, Troponin C	Ara h 1 Ara h 3 Ara h 2 Ara h 6	2S albumins, vicilins, legumins, nsLTPs

### CMA diagnosis and management

**Fig. 2:**
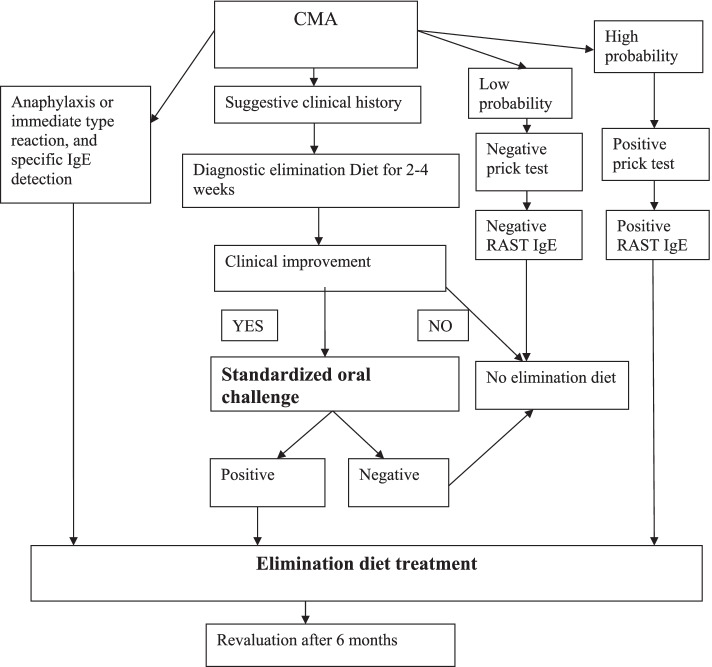
CMA diagnosis and treatment algorithm

Diagnosis of CMA is based on the European Society for Paediatric Gastroenterology Hepatology and Nutrition (ESPGHAN) guidelines [12]. Accordingly, patients with suspected symptoms should undergo an elimination diet for 2–4 weeks; then, if there is a clinical improvement, the diagnosis must be confirmed by a standardised oral food challenge test (Fig. 2). Treatment consists of cow’s milk and derivate elimination diet. After a confirmed diagnosis, exclusive breastfeeding for 4–6 months is the first choice to treat these children. As a second-line treatment option, the elimination of cow milk and milk derivate from the mother’s diet is suggested [13]. Breast milk has demonstrated beneficial effects on immune modulation, promoting the systemic release of IL-10, which plays a central role in the down-regulation of inflammation, and it has been associated with reduced disease severity of CMA-related atopic dermatitis in children [14]. However, there is no conclusive evidence on the protective role of breastfeeding on the onset of specific FAs [15].

When breastfeeding should be not available or contraindicated, special milk formulas are considered the diet substitute for infant nutrition. The special formulas with reduced allergenicity are: extensively hydrolysed formula (eHF), containing oligopeptides with a molecular weight < 3000 Da; partially hydrolysed formula (pHF); amino acid formula (AAF), containing only free amino acids; soy formula (SF); rice hydrolyzed formula (RHF); and other mammalian milk (sheep, goat, camel). The recommended formula is eHF [9]. AAF is recommended only for infants with IgE-mediated CMA at high risk for anaphylactic reactions [16]. Soy formula is not recommended for infants under 6 months of age, as it can induce sensitization to soy proteins [17]. Soy formula can be considered in CMA when eHF are not tolerated, although the possibility of cross-reactivity cannot be excluded [18].

Diet can directly influence organs' growth but could also act as an epigenetic modulator at different levels of immune system function in children suffering from CMA [19–24]. Indeed, a significantly lower incidence of respiratory-associated adverse events and diarrhoea has been found in infants fed with formula with added bovine milk fat globule membrane and bovine lactoferrin [20]. The eHF supplemented with the probiotic Lactobacillus Rhamnosus GG (LGG) seems to reduce the incidence of allergic manifestations and promote the development of oral tolerance in children with IgE-mediated CMA [21]. Moreover, a cohort study on 365 children showed that eHF supplementedwith the probiotic LGG reported the best effect in reducing the incidence of allergic manifestations and improving the immune tolerance acquisition rate compared with the other formulas [24].

Due to their effects on neurodevelopment and immune response, there is also a growing interest in the role of long-chain polyunsaturated fatty acids (LCPUFA) added in milk formulas. The n-3 LCPUFA supplementation during pregnancy has shown a reduced risk of egg sensitization in the first year of life and a reduced risk of developing atopic eczema [22]. Milk formulas with added LCPUFA could influence the risk of developing allergies in children [23]. More recently, in their systematic review and meta-analysis, authors reported that LGG may promote oral tolerance in children suffering from CMA (moderate-quality evidence), as well as facilitating recovery from intestinal symptoms [25]. After 6 months of elimination diet, a revaluation of the child is indicated to consider the possibility of cow’s milk reintroduction. Cow’s milk reintroduction starts with baked milk products and after with fresh cow’s milk (Fig. 1) [12, 16].

### Food introduction time and immunotherapy

Previously, postponing the introduction of allergenic foods to prevent food allergies was suggested. Currently, a new approach has emerged to reduce the incidence of FA. Indeed, the Learning Early About Peanut (LEAP) trial has shown the importance of the early introduction of allergens. In children at high risk for allergy, the early introduction of peanuts in the first year of life prevented the development of peanut allergy [26]. Furthermore, maternal peanuts consumption during breastfeeding combined with the early introduction of peanuts in children’s diet before the first year of age has been associated with the lowest incidence of peanuts allergy [27]. Considerable caution was also adopted in the timing of egg introduction timing, because of its allergenicity. Nevertheless, it has emerged that the egg introduction between the fourth and sixth months of age has been associated with a reduced risk of egg allergy [28].

Allergy to one type of tree nut was often treated with diet avoidance of all tree nuts. However, it has been found that the rate of clinical co-allergy was lower than that of co-sensitisation and not extended to all tree nuts, thus the diet choices could be less selective [29].

Regarding fish allergy, the most common protein that can cause allergy in children is parvalbumin, whose chemical structure changes based on the fish’s species. These differences explain the evidence that a child with a fish allergy might be allergic to some types of fish but not to others [30].

The most promising therapeutic option to induce food tolerance in FA is Food allergen immunotherapy (FA-AIT). It consists of the administration of the culprit allergen, through different routes, in children with a documented FA, at gradually increasing doses with 2-to-4 weeks intervals, followed by a maintenance phase [31, 32]. FA-AIT aims at obtaining the desensitization towards a specific food and developing the immune tolerance to this food protein. FA-AIT has been proven to reduce the risk of potentially life-threatening allergic reactions in the event of accidental ingestion. Oral immunotherapy (OIT) showed an encouraging clinical efficacy with estimated desensitization rate at around 90%, despite adverse events linked to the allergen intake have been documented. Thus, to limit the incidence of these severe adverse reactions, it has been recently suggested OIT administration in with anti-IgE antibodies [33]. Epicutaneous immunotherapy (EPIT) is also possible, and it consists of applying a patch containing a food allergen on the children’s skin. However, OIT seems more effective than EPIT in treating peanut allergy [34]. The European Academy of Allergy and Clinical Immunology (EAACI) has published clinical guidelines on AIT for IgE-mediated FAs, recommending OIT for persistent cow’s milk, hen’s egg and peanut allergies in children from 4 -5 years of age [35].

Currently, food avoidance remains the first-line treatment strategy for treating FA, and the early introduction of food seems to be a promising prevention strategy in developing immune tolerance. New immunotherapy strategies will change the management of FAs.

### Diet treatment in cystic fibrosis

CF is an autosomal recessive disease caused by genetic mutations that interest the CF transmembrane conductance regulator (CFTR) gene. The incidence of CF is around one in 3500 white births in Europe [36]. It is a multisystem disorder that not only affects the lung but it may also involve other organs, like the gastrointestinal tract, with a significant impact on the patient’s nutritional status, mainly secondary to pancreatic insufficiency with consequent malabsorption and risk of malnutrition. Nutrition management is a fundamental element in CF therapy, and a high-fat diet is the basis of this treatment. The updated guidelines on nutrition management of patients with CF also recommend with a high-grade of evidence nutrition education and behavioural counselling for families of infants with CF [37].

Table 2 shows the energy need of CF patients compared to healthy people and the timing of monitoring according to the patient’s age (Table 2).

**Table 2:** Nutritional status needs and monitoring in CF

Age	Energy need	Growth monitoring	Timing of monitoring	PERT monitoring
** < 2 years**	110–200% than same age healthy population	Length for age percentiles	Clinical visit every 1–2 months	Every clinic visit
**2–18 years**	110–200% than same age healthy population	BMI	Clinical visit every 3 months	Every 3 months
**> 18 years**	110–200% than same age healthy population	BMI	Clinical visit every 3–6 months	Every 6 months

*PERT* Pancreatic Enzyme Replacement Therapy, *BMI* Body Mass Index

In children with CF, the high-fat diet combined with microspheric pancreatic enzyme replacement therapy improved outcomes and life expectancy [38]. Accordingly, due to the link between nutritional status and clinical outcome in these patients, children with a lower body mass index (BMI) had an increased incidence of severe lung disease compared to those with a normal BMI. An increased BMI that involves only the fat tissue can also be associated with a worse disease prognosis [39]. The use of age-appropriate BMI-related thresholds is recommended to decide when nutrition interventions are necessary [37].

The relationship between a good nutritional status and a better lung function has been well-established for some years [40]. Infants with CF should receive human milk like healthy infants, since the ones who were breastfed had a better lung function and fewer infections than children who were not breastfed [41]. A standard infant formula could be provided when human milk is unavailable. Infants newly diagnosed with CF fed with a protein hydrolysate formula showed the same energy intake, growth velocity and nutritional status compared with those fed with conventional formula [42]. A retrospective cohort study of preterm CF infants’ growth in the first 2 years of life evidenced that late preterm infants with CF were at higher risk of being below the 10th percentile for weight-for-length at 2 years of age compared with term infants with CF, showing this subgroup of patients need for nutritional attention [43]. Table 3 reports clinical trials investigating different nutritional management in CF (Table 3).

**Table 3:** Clinical trials of nutrition interventions on children with CF

Author	Type of Study	N. of children	Test	Time point	Results
Zahra Gorji et al. Diabetes Metab Syndr. 2020 [44]	Randomized controlled trial	44 CF children and adolescents	Randomized to receive for 3 months either a high fat, high-calorie diet (*n* = 22) or a low glycemic index/high fat, high-calorie diet (*n* = 22)	Lipid profiles, insulin, fasting blood glucose, and glycated hemoglobin at baseline and after the intervention	Adherence to a low glycemic index/high fat, high-calorie diet can improve glycemic indices
Virginia A. Stallings. Plos One. 2020 [45]	Randomized controlled trial	66 with baseline CFA	A three-month treatment with a novel oral structured lipid supplement	Baseline and 3-month evaluations, CFA and height, weight and BMI Z-scores	Subjects in low CFA subgroup had improved CFA (p = 0.002), and reduced stool fat loss
L Ellis J Pediatr. 1998 [42]	Clinical trial	23 CF infants	Randomized to receive a hydrolysate formula or a cow's milk-based formula	Monitored at 1 month and then every 3 months for 1 year	Same energy intake, growth velocity and nutritional status with each formula

*CFA* Stool Coefficient of Fat Absorption, *BMI* Body Mass Index, *CF* cystic fibrosis, *N* number

GERD is a common comorbidity among patients with CF with a high prevalence also in young patients, and it is linked to an increased number of infectious pulmonary exacerbations. A higher prevalence of extra-oesophageal symptoms generally characterizes GERD in CF patients. GERD may affect respiratory function, suggesting that early detection of the disorder and a targeted treatment based on proton pump inhibitors (PPIs) could improve the clinical outcome of these patients [46, 47].

### Malnutrition prevention in CF

Malnutrition is more common in CF children with a documented exocrine pancreatic insufficiency. Pancreatic enzyme replacement therapy (PERT) is considered the main treatment in the event of impaired exocrine pancreatic function [38, 48, 49].

Despite the importance of an appropriate dietary management, some children and adolescents with CF show a low adherence to the dietary recommendations and may end up suffering from malnutrition, which can compromise the immune system and lung function, as well as increasing the risk of respiratory infections [50]. A child with CF needs 10–15% more energy intake than a healthy child; accordingly, the nutritional management of CF patients includes a high-calorie/high-fat diet, added to PERT, and vitamin and mineral replacement. The high-fat diet has improved the nutritional status prolonging survival. The high-caloric diet includes a high-fat intake, which represents 35%–40% of the daily energy requirements, and it is necessary to cover the high-calorie demand of these patients, even throughout the promotion of the use of high-calorie fat additives to foods and beverages [51]. ESPEN guidelines recommend adjusting energy intake to avoid obesity, considering the energy intake adapted by age, and performing regular clinical re-evaluations of patients taking oral nutritional supplements to determine whether the patient should continue taking them [37].

The adherence to a low glycaemic index, high fat, high-calorie diet can improve glycaemic indices in children and adolescents with CF compared to the high fat, high-calorie diet [44]. When an oral supplementation with pressurized whey proteins is chosen, there is an increase in intracellular glutathione levels, improving the inflammatory status of patients with CF [52]. A randomized controlled trial (RCT) conducted on CF children with pancreatic insufficiency showed an improvement in fat absorption and a better growth after treatment with a novel oral structured lipid supplement [45]. ESPEN guidelines strongly recommend a nutrition intervention that comprises a full review of nutrition status and PERT, suggesting investigating any underlying medical conditions that could compromise health status. A polymeric enteral tube feeding could be considered only when oral interventions have failed to obtain an acceptable rate of growth and nutritional status [37].

In summary, managing patients with CF need a multidisciplinary approach, since diet and nutrition intake significantly impact the disease course and susceptibility to severe infections. Before the new frontier of the promising channel modulators, nutritional therapy, based on a high calorie/high-fat diet and PERT in combination with regular follow-up, has changed the natural history of CF patients, and, to date, it continues to represent the primary option to improve survival and quality of life (QoL) in CF.

### Nutrition and diet in inflammatory bowel disease

Ulcerative colitis (UC) and Crohn’s disease (CD) are the most common IBD. UC is a chronic disease featured by diffuse rectal and colic mucosa inflammation, which involves the rectum in 95% of cases. It can be extended continuously to more proximal parts of the large intestine. The classic clinical symptom of UC is bloody diarrhoea, and the clinical presentation is characterized by periods of remission and exacerbation [53]. CD is a chronic inflammatory gut disease involving all gastrointestinal tracts. It generally interests the ileocolic tract with a complete colon wall layers inflammation, which is not continuously extended differently from UC. The most common symptoms of CD are abdominal pain, diarrhea and weight loss [54]. Diagnosis of IBD is mainly histological [55]. During the last years, the incidence of IBD has increased, suggesting the possible pathogenic role of environmental factors. Among these, the influence of diet has been revaluated.

The so-called "Western diet” and some dietary constituents, like fat, seem to have a proinflammatory effect that may promote the onset of IBD in genetically susceptible individuals [56]. Conversely, a prospective study has shown that a long-term intake of dietary fibres, particularly fibres from fruit, was protective against the risk of development of CD [57]. A decreased risk of developing IBD was observed with a diet rich in fruit and vegetables and n-3 fatty acids, which is therefore recommended in these patients [58]. Breastfeeding has been also associated with a lower risk of IBD for its protective role against early onset IBD (OR, 0.69; 95% CI, 0.51–0.94; *p* = 0.02) [59]. A case–control study performed on 1291 patients affected by IBD reported a protective role of breastfeeding against IBD with a prolonged response effect [60]. Similarly, Hansen et al. showed that breastfeeding for more than 6 months decreased IBD risk [61]. A diet rich in sweets was also positively associated with a higher risk of UC, while the intake of vitamin C was negatively related to the risk of UC[62].

### Nutritional treatment

Appropriate nutritional treatment is needed to prevent malnutrition and growth restriction in patients with IBD, since malnutrition has been associated with worsened prognosis, QoL, and increased risk of complications. The protein requirement is higher in active IBD, with an increased intake of nutrients. When the disease is active, and patients are treated with steroids, serum calcium and 25(OH) vitamin D should be monitored and supplementation is required to prevent low bone mineral density [58]. Patients with IBD must receive an individualised nutritional approach because they are at risk of malnutrition due to insufficient intake, malabsorption and protein-losing enteropathy or metabolic disturbances induced by the disease and treatments, in particular corticosteroids [63]. Enteral nutrition (EN) is considered safe and can provide the necessary nutritional intake [64]. However, EN should be considered in patients with a functional gastrointestinal tract but who cannot swallow safely. To date, there is no evidence of changes in the inflammatory response in UC patients with artificial nutritional support [65]. For the European Crohn's and Colitis Organization (ECCO)/ESPGHAN guidelines, exclusive enteral nutrition (EEN) for 6–8 weeks is considered the first-line treatment for induction therapy in children and adolescents with an active CD for its excellent safety profile [66]. Polymeric formulas, consisting of non-hydrolyzed proteins, carbohydrates, and fat, are less expensive and are considered more palatable than elemental formulas; they may be the best choice if the oral route is feasible [62]. The specific carbohydrate diet aims at reducing bowel inflammation, restricting the introduction of carbohydrates to monosaccharides and few polysaccharides. It seems to improve symptoms, though data are limited. Other dietary regimens have been suggested, such as an anti-inflammatory diet and CD exclusion diet, whose efficacy needs to be further assessed by larger studies [67]. Parenteral nutrition (PN) is indicated when the bowel is obstructed or not functional or there is no possibility to place a feeding tube beyond the obstruction. It is required in patients with short bowel when there is severe malabsorption of nutrients and the enteral feeding is not sufficient [68].

If the colon and rectum are surgically removed, they are replaced by a pouch with the rectum's function. Pouchitis, which occurs in around 50% of patients, is the inflammation of this intestinal tract. Pouchitis is common in patients with a low intake of antioxidant substances, making the mucosal pouch exposed to inflammatory molecules that can cause oxidative stress, leading to chronic inflammation. This event is probably the most important pathogenetic mechanism of pouchitis [69]. The slowed faecal transit is also important in the pathogenesis of this disease because it can create immunologic reactivity. Probiotics have been suggested to prevent inflammation of the pouch [70, 71]. Mainly, two double-blind placebo-controlled trials have investigated a high-potency probiotic mixture. The authors reported the positive effect of a high-potency probiotic mixture in patients with chronic pouchitis on the duration of remission [72]. Other authors showed the protective effect of enteral supplementation of inulin against pouch inflammation [73].

Differently, omega-3 fatty acid supplementation in patients with UC does not affect the maintenance of UC remission, while a positive effect was observed in an RCT on children with CD [74, 75]. Regarding the use of probiotics in the induction and maintenance of remission in UC, Miele et al. demonstrated the efficacy and role of a high-potency probiotic mixture in maintaining remission in active UC [76]. Another intriguing effect of using high-potency probiotic mixture is reducing the disease activity index. The critical role of probiotics “E. coli Nissle 1917” and the high-potency probiotic mixture was the maintenance of remission in children with mild and moderate UC [77]. Finally, according to ECCO guidelines, a high-potency probiotic mixture can be used to maintain remission after antibiotic therapy and prevent the inflammation of the pouch in paediatric patients [78]. Overall, the diet has emerged as an important player in the pathogenesis, progression and treatment of IBD. In paediatric patients, an individualised nutritional approach based on EEN, avoidance of proinflammatory nutrients in CD and probiotic supplementation in UC has showed promising results as first-line or adjunctive therapy, though long-term adherence is challenging. RCTs are required to confirm their effectiveness [79]. Table 4 summarizes studies on IBD dietary management.

**Table 4:** Trials on IBD diet management

Author	Type of Study	N	TEST	Time points	Results
Gnocchetti et al. 2000 [71]	Randomized controlled trial	40	VSL#3 6 g/day or an identical placebo for 9 months	Clinically every month; endoscopically and histologically every 2 months	Oral administration of probiotics is effective in preventing flare-ups of chronic pouchitis
Welters et al. 2002 [73]	Randomized, double-blind, crossover study	20	24 g of inulin or placebo daily during three weeks	stools were analyzed after each test period for pH, short-chain fatty acids, microflora, and bile acids. Inflammation was assessed endoscopically, histologically, and clinical	enteral inulin supplementation leads to a decrease of inflammation-associated factors and a reduction of inflammation of pouch mucosa
Sakamoto et al. 2005 [62]	Case–control study	239	Questionnaire	//	sweets were positively associated with UC
Miele et al. 2009 [76]	Prospective, 1-year, placebo-controlled, double-blind study	29	Randomized to receive VSL#3 (*n* = 14) or an identical placebo (*n* = 15)	1 month, 2 months, 6 months, and 1 year after diagnosis valuated with Lichtiger colitis activity index	Remission in 13 patients treated with VSL#3 and IBD therapy and in 4 patients treated with placebo and IBD therapy (*P* < 0.001)
Gearry et al. 2010 [60]	Population-based case–control study	1291	Questionnaire	//	childhood vegetable garden was protective against IBD as was having been breastfed with a duration-response effect
Hansen et al. 2011 [61]	Case–control study	123	Questionnaire	//	Being breastfed > 6 months decreased the risk for IBD
Arie Levine et al. 2019 [66]	Prospective trial	74	A group CDED plus formula for 6 weeks followed by CDED with PEN from weeks 7 to 12; a group: EEN for 6 weeks followed by free diet, 25% PEN	Dietary tolerance, ITT remission at week 6 and corticosteroid-free ITT remission at week12	CDED plus PEN induced sustained remission

*CDED* Crohn's Disease Exclusion Diet, *N* number

### Diet treatment in short-bowel syndrome (SBS)

Intestinal failure *can* be defined as the reduction of functional gut mass that provokes inadequate digestion and absorption of nutrients and fluid and a consequent need for PN [80]. The causes of this condition could be intestinal obstruction, dysmotility, surgical resection, congenital defects, or disease with loss of absorption [80]. This large group of diseases includes the common specific gut condition called SBS, caused by surgical resection, congenital defect, or diseases associated with loss of absorption gut capacity. The pathophysiological mechanism is the inability to maintain energy, fluid, electrolyte, macronutrient, and micronutrient needs in a regular diet [81–83]. The main characteristics are fast intestinal transit associated with diarrhoea and malabsorption [81–83]. Three types of SBS have been defined based on anatomical criteria: 1) end-jejunostomy when ileum and colon are removed; 2) jejuno-colonic anastomosis when all or most of the ileum is removed; 3) jejuno-ileal anastomosis when ≥ 10 cm of the terminal ileum and the entire colon are preserved [84]. In the acute phase, immediately after intestines resection, PN allows to obtain the necessary energy requirements, through the administration of nutrients by the intravenous route. Some patients will require PN for a limited period of life, while others, like patients with jejunostomy, could remain dependent on parenteral support, and may be candidates for bowel transplantation, if they develop complications such as PN associated liver disease [83, 85]. This condition can be prevented by using lipid emulsion containing fish oil rich in omega-3 that promotes the bile flow and may regulate the inflammatory process. In this phase, a central catheter must be placed in a central vein, if long-term PN is needed. PN contains all nutrients, both macro and micronutrients [86–91]. During the adaptation phase, the remaining bowel increases fluid and nutrient absorption through muscular hypertrophy of intestinal walls and mucosal hyperplasia [86–91]. In this critical phase, EN can provide the intraluminal nutrients with a stimulatory effect on the epithelial cells and consequently increase the production of trophic hormones promoting mucosal regeneration [86–91]. Enteral feeding also increases pancreatic and biliary secretions [86–91]. Nevertheless, patients with jejunostomy do not undergo the adaptation phase because of the extension of resection. When residual jejunum is less than 75 cm, long-term PN may be needed [92].

After surgery, EN should be administered early with a "minimal enteral feeding" [86–91]. If it is possible, it is preferable to start diet treatment with small breast milk volume, which contains a lot of essential factors for bowel growth and adaptation: nucleotides, immunoglobulin A, leucocytes, glutamine, growth hormone and epidermal growth factor, which are responsible for the mucosal barrier modulation [86–91]. The administration of a continuous slow EN gradually improves intestinal function. This process is mediated by the saturation of carrier proteins. When half of the total energy requirements are provided by continuous EN and PN, EN management may be switched to an intermittent administration [86–91]. EN is not feasible in all patients with SBS, and long-term PN is needed if absorption is inferior to a third of the oral energy intake, if energy requirements are high and absorption is 30–60%, or if increasing oral/enteral nutrient intake causes remarkable diarrhoea or a large volume of stomal output [92]. During the adaptation phase, the bowel is more permeable, and children with SBS are at risk of developing FA [93]. Different types of diet can be used in this condition: natural diets, dairy diets (using breast milk), dietary supplements with synthetic preparation, elemental and semi-elemental diets with hydrolysed proteins and carbohydrates, modular diets, characterized by low cost and obtained by a mixture of nutrients variable during the time [93, 94]. In accordance with the most recent literature, human milk is the first diet step for infants in SBS. If breast milk is not available, polymeric formulas are recommended [93, 94], and, among them, hydrolysed formulas are considered not superior compared with AAF in these patients. Therefore, both formulas could be used as a second step diet treatment [93, 94]. When it is possible, starting bottle-feeding stimulates the suck and swallow reflex; thus solid food can be introduced at the age of 4 to 6 months [93, 94]. If the colon is intact, soluble fibres can be added to the diet, being their use associated with a longer transit time and higher nitrogen absorption, so minimizing the side effects of treatments [95, 96]. In older children, blended family foods can be administered into an enteral feeding tube improving clinical outcomes such as gagging, vomiting and tolerance [97, 98]. During the treatment, it is important to monitor stool consistency and volume, weight, and hydration status of the mucosae. Furthermore, blood tests should be performed to evaluate electrolytes and acid–base homeostasis [83]. Table 5 reports the suggested nutritional approach to SBS and the route of administration.

**Table 5:** Nutrition in short-bowel syndrome (SBS)

AGE	Type of nutrition	Mode of administration
Neonate	Breast milk/polymeric HF/AA	EN, when is possible oral
Infant (4–12 Months)	breast milk, Puree, Solids (e.g., bread) EN: Polymeric/HF/AA	Oral and partial enteral
Child (Solid Foods)	Solid foods EN: Polymeric/HF/AA	Prefer oral, optionally partial enteral

*HF* polymeric hydrolysed formula, *AA* free amino acid formula, *EN* enteral nutrition

### Diet management of eosinophilic esophagitis

EoE is due to a chronic immune-mediated inflammation that causes alteration and dysfunction of this intestinal tract [99]. Regarding its pathogenesis, an interaction between genetic and environmental factors, including allergic sensitization and epigenetic phenomenon, has been suggested [99]. EoE is considered a chronic atopic disease, as patients with eoE present other atopic diseases such as allergic rhinitis, IgE-mediated FA, asthma, and atopic dermatitis. The local inflammation of the epithelium is generally mediated by Th2 response with IgE and non-IgE mediated reaction to environmental and food allergens. Commonly, the trigger is food that typically causes non-IgE mediated Th2 inflammation. There is limited evidence about aeroallergens triggers which may be instead important in young adults. During the last years, it has been reported some cases of EoE developed after OIT used as a treatment of IgE-mediated FA [100]. After GERD, EoE is considered the main cause of dysphagia among children and young adults in Europe and North America [101]. The diagnosis of EoE is histological, and it is obtained from six oesophageal mucosal biopsies, with a diagnostic threshold of > 15 eosinophils/high power field with a sensitivity of 100% and a specificity of 96% [99, 102].

A trial with PPI at high dosage for 8-12 weeks, dietary modifications and steroids are considered the first-line therapy in children with EoE [99]. There are three kinds of elimination diets: elemental diet, targeted by allergy testing diet, and empiric diet [102]. The first study to use exclusively an elemental diet demonstrated that all patients had normalized oesophageal histology after treatment [103]. It has been found that an elemental diet can be efficacious in up to 90% of children with EoE [102]. A review showed that the use of AAF diminished eosinophil count, inducing remission in 75%‐100% of children. Based on these data, AAF is considered superior compared with the food elimination diet (FED) [103–105]. However, an elemental diet with AAF is used only to treat persistent active EoE, refractory to other conventional drugs and/or empiric diets because of poor palatability, use of nasogastric tubes, lack of adherence, psychological impact, and high costs [103–105].

Cow’s milk, wheat/gluten, egg, and soy/legumes are the most frequently involved foods in the onset of EoE. Nuts and fish/sea-food rarely trigger EoE [106–108]. In paediatric patients with EoE, dietary treatment is generally empirical, based on the elimination of the child's diet of the most common foods implicated in immediate hypersensitivity. The six Food Elimination Diet (FED), including milk, wheat, egg, soy, nuts, and fish/seafood was firstly described by Kagalwalla et al., who reported clinical and histological remission in 74% of children affected by EoE [109]. These results were confirmed in other paediatric studies [109–111]. To optimize dietary and endoscopic management, a step-up approach was investigated using a two or four FED [109]. With a four FED, the remission rate was about 64% [109]. Interestingly, 45% of patients had cow’s milk as unique trigger of EoE, so excluding cow’s milk and wheat could be sufficient. This step-up approach was proposed in a study involving 130 paediatric and adult patients. The first step was the two FED-milk and gluten. If the patient was not responder, four FED was offered and, finally, six FED. This approach allowed the reduction of endoscopic procedures and time to diagnosis [108]. However, another study reported a relapse following the reintroduction of the triggering foods four years after the elimination diet [109]. Table 6 reports trials on different FED in patients with EoE (table 6). At this time, in clinical practice, therapy is modulated on the single patient case, and when the disease is in remission, slow reintroduction of eliminated food is recommended. Milk, soy, wheat, egg, and meats may be reintroduced once a week and, in each reintroduction, esophagogastroduodenoscopy (EGD) should be repeated two months after the reintroduction [109]. It is also suggested to monitor and manage micro- and macro-nutrients dietary deficiencies that can occur in patients under FED. Physicians and caregivers should be correctly informed about this medical problem. The recent increase in the incidence of EoE, especially in Western countries, has raised the need of studies about the long-term efficacy of dietary treatment, especially after the reintroduction of each food.

**Table 6:** Studies on different dietary approaches in EoE

Author, type of study, population	Diet, (N)	Histologic remission rate (%)	Number of foods identified	Most common food identified
Kagalwalla 2011, Retrospective single centre, children	SFED *N* = 46	74%	1: 72% 2: 8% > 2: 8%	Milk 74% Wheat 26% Eggs 17%
Kagalwalla 2017, prospective multicenter children	FFED *N* = 78	64%	1: 64% 2: 20% > 2: 16%	Milk 85% Egg 35% Wheat 33% Soy 19%
Molina-Infante 2018, prospective multicenter, adult and children	TFED *N* = 130 (pediatric = 25)	43%	1: 58% 2: 33% > 2: 9%	Milk 81% Wheat/gluten 43% Egg 15% Legumes 9%

*SFED* six-food elimination diets, *N* number, *FFED* 4-food elimination diet, *TFED* testing–directed food elimination

### Diet treatment in infant with gastroesophageal reflux disease

Gastroesophageal reflux is the transit of stomach material in the oesophagus and may be considered a physiologic process [110]. GERD is defined as the reflux that produces significant symptoms and may cause complications, such as reflux esophagitis, strictures, respiratory complications, failure to thrive, Barrett oesophagus, and oesophageal adenocarcinoma [111]. Different conditions have been associated with an increased risk of GERD: hiatal hernia, neurodevelopmental disorders, cystic fibrosis, epilepsy, congenital oesophageal disorders, asthma, and prematurity [112]. Milk protein sensitivity symptoms can be sometimes difficult to differentiate from GERD [113]. Indeed, a prospective study showed the possibility of a co-existence of milk protein sensitivity and GERD [114]. Hence, infants with persistent symptoms may benefit from 2 to 4 weeks use of eHF [115]. GERD symptoms can be improved by eating changes, especially in infants, such as reducing feeding volumes. Moreover, in nations with good economic and social standards, where infants are overfed, smaller and more frequent eating might reduce reflux episodes, and represent the first therapeutic approach [111]. In healthy infants with a persistent regurgitation associated with poor weight gain, thickened formulas (TFs) are suggested, since adding a thickening agent, such as cereal starch or carob locust bean gum flours, increases milk viscosity and reduces gastric reflux [116–118]. Anti-reflux formulas have a verified composition with 2 g/100 mL for starch and 1 g/100 mL for carob bean gum; thereby, their caloric content is similar to standard formulas. Interestingly, pre-treated gelatinized starch presents a low viscosity and thickens only in the stomach when it comes into contact with acid pH [119]. TFs can be homemade to overcome their high costs, but they show a heterogeneous composition, with increased osmolality and extra caloric intake. TFs and anti-reflux formulas can allow a gain in weight when compared to standard formulas [120]. Other improved outcomes include sleeping, irritability, cough and choking [119]. Currently, no thickening agent can be considered better than another. Episodes of vomiting and regurgitation have been often used to measure the efficacy of the therapeutic intervention. Hence, three studies reported a reduction in the number of episodes of regurgitation per day [121–123] and two studies showed a reduction in the number of episodes of vomiting per day [121, 124]. Ostrom et al. demonstrated that soy formula with added fibres was superior to cow’s milk in terms of reduction of regurgitation [122].

Infants with physiological GER may receive breastmilk, and mothers should be encouraged to continue breastfeeding. If reflux becomes significant, mother’s milk can be supplemented with xanthum gum or carob bean, while cereals are not recommended because the amylases of human milk degrade it. Specifically, carob bean can be used in infants after 42 weeks gestation and xanthum gum is allowed in 1-year old infants or older [125].

Regurgitation can often be the only symptom of CMA, so infants with suspected CMA should receive eHF. AAFs are indicated for patients with severe symptoms [126]. Children with suspected CMA fed with AAFs showed fewer reflux events than the cow’s milk feeding [127]. ESPGHAN guidelines recommend against the use of soy-based infant formula [128]. 10 – 15% of infants with CMA will also become allergic to soy [129]. Rice hydrolysates are commercially available and can reduce regurgitation [130]. Regarding the use of L. Reuteri added to milk formula, a trial demonstrated that the mean number of regurgitations per day was fewer than the placebo group [131].

In children with CF the prevalence of GERD is increased with a prevalence of acid reflux. Several studies highlighted a correlation between GERD and more severe lung disease with increased respiratory exacerbations. In children with CF, GERD has correlated with early *Pseudomonas aeruginosa* and *Staphylococcus aureus* infection and decreased pulmonary function. The latter may be due to micro-acid aspirations that increase inflammation and surfactant damage, resulting in micro-atelectasis and infection. The role of GERD in the decline of pulmonary function is also important after lung transplantation. Accordingly, manometry, pH monitoring and bronchoscopy with bronchoalveolar lavage fluid BALF for detection of pepsin or bile acids are indicated in this cluster of patients.

To summarize, if patients are not responders to conventional GERD therapies, the paediatrician can use eHF or AAF based on the symptom’s severity (Table 7). The duration of a non-pharmacological therapy should be at least two weeks, before changing the therapeutic strategy in the event of non-response.

**Table 7:** Milk diet in infant with GERD

Type of milk	Indication	Composition	Characteristics	Costs
TFs	Persistent regurgitation, poor weight gain, important symptoms	2 g/100 mL for starch or 1 g/100 mL for carob bean gum addicted to SF	It increases milk viscosity reducing the gastric reflux and other symptoms	** + **
EEF	Suspected CMPA (second choice)	Peptides < 5000 Da	It increases gastric emptying speed	** + + **
AAF	severe symptoms (selected cases)	Free amino acids 2,1 gr/100 mL	It reduces reflux events	** + + + **
HRPFs	Alternative to EEF if CMPA is suspected	Lactose-free, Addition of free Amino-Acids: Lysin Threonine Tryptophan	Well tolerated, appropriate growth pattern	** + **

*TFs* thickened formulas, *EEF* extensively hydrolysed formulas, *AAF* amino acid formulas, *HRPFs* hydrolysed rice protein formulas

## Discussion

Nutrition is a crucial environmental factor that can be able to affect, directly or indirectly, human health, through the effect of metabolites on the gut microenvironment. Since the first months of life nutritional choices may have significant implications on infant’s growth and health. Human milk, containing all nutrients and some other factors that can modulate the immune system, is considered the best and complete food for infants’ nutrition. Hence, exclusive breastfeeding is recommended by WHO guidelines until the age of six months [1]. Paediatricians play a key role in promoting breastfeeding, and alternatives to human milk should be chosen only in specific pathological conditions or if human milk is not available. Indeed, breastfeeding has been associated with reduced risk of development of IBD and better lung function and outcome in CF, and it is the first-line treatment of CMA. Special formulas can be considered to treat CMA, EoE, and GERD.

During weaning, the timing of the introduction of solid foods in infants’ diet has been long debated. Recent evidence supports that the early introduction of allergenic foods reduces the risk of FA compared with delayed introduction. If FA develops, the mainstay of treatment is the avoidance of the culprit food and it can be also applied in EoE. However, FA-AIT is emerging as a promising strategy to induce tolerance against allergenic food.

Some diseases expose patients to the risk of malnutrition and need specific dietary management, like the high calorie/high fat diet and PERT in CF and polymeric formulas and the specific carbohydrate diet in CD. The former has showed to improve outcome and survival in CF. The latter has been used to induce and maintain remission in CD. The role of probiotics has also been increasingly recognised, since impaired gut microbiota has been suggested to be involved in the pathogenesis of some gastrointestinal disorders. Accordingly, the supplementation with probiotics may have a role in the induction and maintenance of remission in UC and in reducing symptoms of GERD.

When neither the oral nor the enteral route is available, like in SBS, PN is needed for a variable length of time based on the severity and extension of intestinal dysfunction.

## Conclusions

In conclusion, we provided an overview of dietary management in different diseases affecting the gastrointestinal tract. An appropriate dietary management combined with growth monitoring should be the first therapeutic approach and can allow to prevent and treat diseases, often avoiding pharmacological treatment that can be potentially burdened by side effects and disease complications. However, if some dietary treatments like human milk and PERT in CF has been proven effective, others need to be further investigated in RCTs in a larger population. Regarding paediatricians and physicians, they should be aware of their essential role in promoting breastfeeding, healthy nutritional habits, the appropriate timing of introduction of solid foods during weaning and managing specific disorders with appropriate nutritional treatment.

## Data Availability

The datasets used and/or analysed during the current study are available from the corresponding author on reasonable request.

## Abbreviations

AAF: Amino acid formula
BMI: Body mass index
CD: Crohn’s disease
CF: Cystic fibrosis
CFTR: Cystic fibrosis transmembrane conductance regulator
CMA: Cow's milk proteins allergy
EAACI: European Academy of Allergy and Clinical Immunology
ECCO: European Crohn's and Colitis Organization
EEN: Exclusive enteral nutrition
EGD: Esophagogastroduodenoscopy
eHF: Extensively hydrolysed formula
EN: Enteral nutrition
EoE: Eosinophilic esophagitis
EPIT: Epicutaneous immunotherapy
ESPEN: European Society for Clinical Nutrition and Metabolism
ESPGHAN: European Society for Paediatric Gastroenterology Hepatology and Nutrition
FA-AIT: Food allergen immunotherapy
FED: Food elimination diet
GERD: Gastroesophageal reflux disease
IBDs: Inflammatory bowel diseases
LCPUFA: Long-chain polyunsaturated fatty acids
LEAP: Learning early about peanut
LGG: Lactobacillus rhamnosus GG
OIT: Oral immunotherapy
PERT: Pancreatic enzyme replacement therapy
pHF: Partial hydrolysed formula
PI: Pancreatic insufficiency
PT: Parenteral nutrition
RCT: Randomized controlled trial
RHF: Rice hydrolyzed formula
SBS: Short-bowel syndrome
SF: Soy formula
UC: Ulcerative colitis
